# In-Depth Investigation of Archival and Prospectively Collected Samples Reveals No Evidence for XMRV Infection in Prostate Cancer

**DOI:** 10.1371/journal.pone.0044954

**Published:** 2012-09-18

**Authors:** Deanna Lee, Jaydip Das Gupta, Christina Gaughan, Imke Steffen, Ning Tang, Ka-Cheung Luk, Xiaoxing Qiu, Anatoly Urisman, Nicole Fischer, Ross Molinaro, Miranda Broz, Gerald Schochetman, Eric A. Klein, Don Ganem, Joseph L. DeRisi, Graham Simmons, John Hackett, Robert H. Silverman, Charles Y. Chiu

**Affiliations:** 1 Department of Laboratory Medicine, University of San Francisco, San Francisco, California, United States of America; 2 University of California San Francisco-Abbott Viral Diagnostics and Discovery Center, University of California San Francisco, San Francisco, California, United States of America; 3 Cleveland Clinic, Cleveland, Ohio, United States of America; 4 Blood Systems Research Institute, San Francisco, California, United States of America; 5 Abbott Laboratories, Abbott Park, Illinois, United States of America; 6 University Medical Center Hamburg-Eppendorf, Hamburg, Germany; 7 Emory University School of Medicine, Atlanta, Georgia, United States of America; 8 Novartis Institutes for Biomedical Research, Emeryville, California, United States of America; 9 Department of Biochemistry and Biophysics, University of California San Francisco, San Francisco, California United States of America; 10 Howard Hughes Medical Institute, Chevy Chase, Maryland, United States of America; 11 Department of Medicine, Division of Infectious Diseases, University of California San Francisco, San Francisco, California, United States of America; Burnet Institute, Australia

## Abstract

XMRV, or xenotropic murine leukemia virus (MLV)-related virus, is a novel gammaretrovirus originally identified in studies that analyzed tissue from prostate cancer patients in 2006 and blood from patients with chronic fatigue syndrome (CFS) in 2009. However, a large number of subsequent studies failed to confirm a link between XMRV infection and CFS or prostate cancer. On the contrary, recent evidence indicates that XMRV is a contaminant originating from the recombination of two mouse endogenous retroviruses during passaging of a prostate tumor xenograft (CWR22) in mice, generating laboratory-derived cell lines that are XMRV-infected. To confirm or refute an association between XMRV and prostate cancer, we analyzed prostate cancer tissues and plasma from a prospectively collected cohort of 39 patients as well as archival RNA and prostate tissue from the original 2006 study. Despite comprehensive microarray, PCR, FISH, and serological testing, XMRV was not detected in any of the newly collected samples or in archival tissue, although archival RNA remained XMRV-positive. Notably, archival VP62 prostate tissue, from which the prototype XMRV strain was derived, tested negative for XMRV on re-analysis. Analysis of viral genomic and human mitochondrial sequences revealed that all previously characterized XMRV strains are identical and that the archival RNA had been contaminated by an XMRV-infected laboratory cell line. These findings reveal no association between XMRV and prostate cancer, and underscore the conclusion that XMRV is not a naturally acquired human infection.

## Introduction

In 2006, sequences corresponding to a novel gammaretrovirus named xenotropic murine leukemia virus-related virus (XMRV) were identified in tissue from prostate cancer patients following radical prostatectomy [1]. The discovery of XMRV was accomplished using a broad-spectrum microarray assay (ViroChip) designed to detect all known viruses as well as novel viruses on the basis of sequence homology [1], [2], [3]. The results from this study also revealed an association between the presence of XMRV and patients known to be homozygous for the R462Q variant of RNAse L, a gene previously linked to the hereditary prostate cancer 1 locus [4]. Mutations in RNAse L that impair the apoptotic response to viral infection were postulated to reflect enhanced susceptibility to infection by XMRV and suggested a potential role for the virus in carcinogenesis [5], [6], [7], [8]. Although the initial study reported a link between RNAse L-variant prostate cancer and XMRV infection, most, but not all, subsequent studies have failed to detect such an association [9], [10], [11], [12]. Since this initial discovery, XMRV and MLV-related virus sequences resembling polytropic MLVs (P-MLVs) were also found in patients with chronic fatigue syndrome (CFS) [13], [14].

Subsequent reports have cast doubt on the association of XMRV with prostate cancer or CFS, and indeed on whether XMRV is even found in humans (reviewed in [15]). Moreover, the viral sequences from XMRV-positive patients lacked the level of genetic diversity expected for retroviral infections [1], [14], implying that XMRV may have arisen from sample contamination and not true viral infection. Nearly all follow-up studies using specific PCR have largely failed to confirm the presence of XMRV in either CFS or prostate cancer cohorts [12], [16], [17], [18], [19], [20], [21], [22], [23], [24], [25], [26], [27], [28], [29], [30], [31], [32], resulting in retraction of the initial papers linking XMRV and P-MLVs with CFS [33], [34]. A 2009 study found, unexpectedly, that a common laboratory cell line called 22Rv1, derived from the CWR22 human prostate cancer xenograft, produced high titers of XMRV [35]. This was followed by a study from Garson, *et al.* demonstrating that identical XMRV integration sites were shared between putatively infected prostate tumor tissues and an experimentally infected laboratory cell line [36], [37], further undermining the prospect that XMRV is a genuine human pathogen. Finally, a 2011 study from Paprotka, *et al.* provided strong evidence that XMRV is a 22Rv1-derived laboratory contaminant originating from recombination of two mouse endogenous retroviruses during serial passage of CWR22 in nude mice [38]. The recent demonstration that XMRV and related viruses are not present in the primary prostate tumor tissue from the patient CWR22 lends additional support for this hypothesis [39].

**Figure 1:**
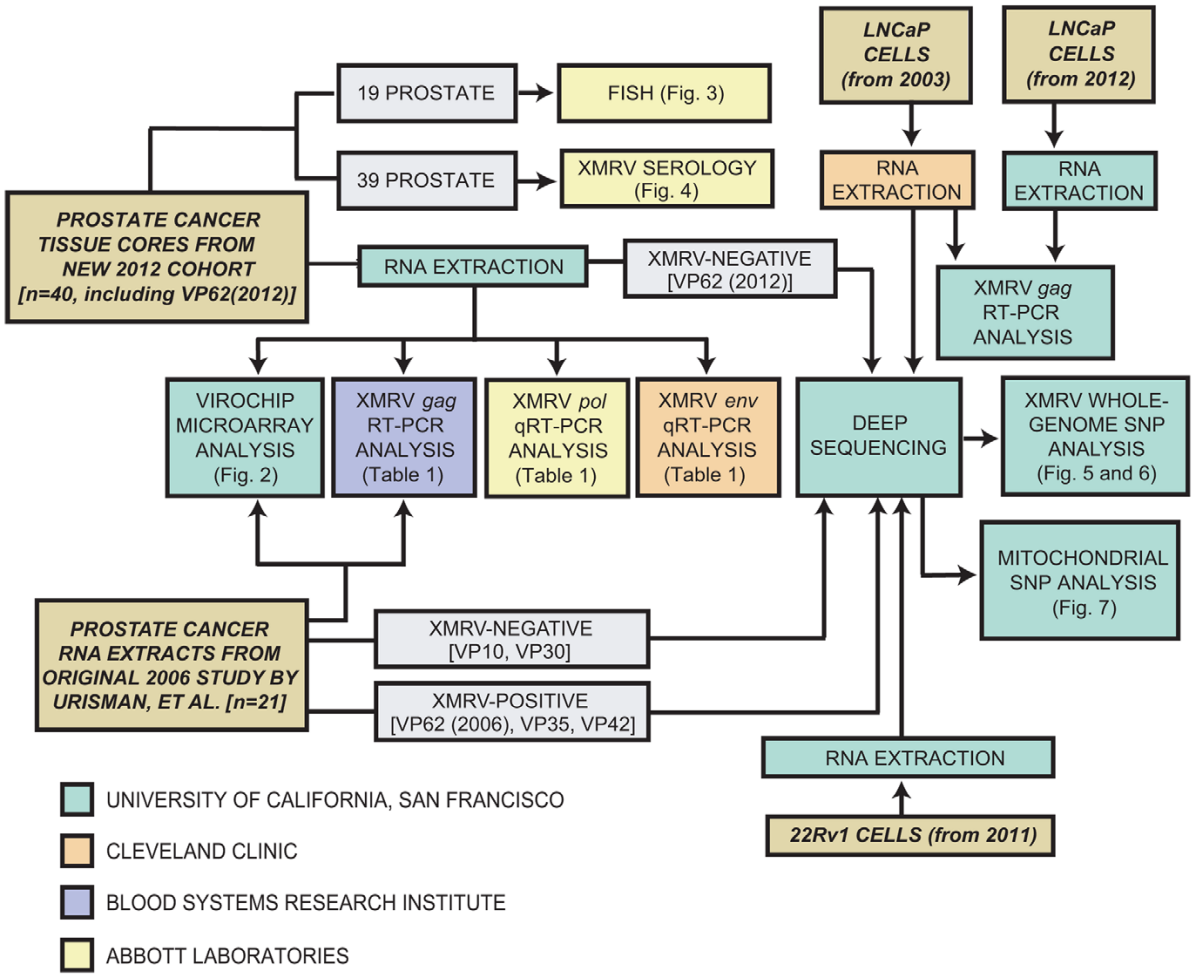
Study Workflow. Colored boxes refer to the research laboratory in which the described analysis was performed. To minimize the risk of PCR amplicon contamination, blinded XMRV-specific PCR testing was performed separately in 3 independent laboratories.

Given the clinical and public health implications of potential XMRV infection in humans, we sought to confirm or refute the association between XMRV and prostate cancer. To date, most of the negative studies have been carried out in CFS and not in prostate cancer, and some have speculated that the original discovery of XMRV may in fact reflect *bona fide* viral infection but that subsequent studies were potentially tainted by mouse genomic contamination and/or widespread circulation of positive control plasmids containing the XMRV infectious molecular clone VP62 [40], [41], [42]. We present here an in-depth investigation using samples taken from both a prospective cohort of 39 new prostate cancer patients and the original 2006 Urisman *et al.* study in which XMRV was discovered [1]. In particular, archival radical prostatectomy tissue from patient VP62, used to generate the VP62 clone employed in nearly all downstream molecular and cellular studies of XMRV [43], was available for analysis. ViroChip microarray, PCR, fluorescence *in situ* hybridization (FISH), serological, and deep sequencing analyses were performed in 4 different laboratories (Fig. 1). The combined findings are most consistent with XMRV-associated laboratory contamination of the prostate cancer tissues analyzed in original 2006 Urisman, *et al.* study [1] and provide a clear explanation as to how such contamination could have occurred.

## Results

### XMRV Not Detected in Newly Collected Prostate Cancer Samples and Archival VP62 Tissue by Microarray Screening

**Figure 2:**
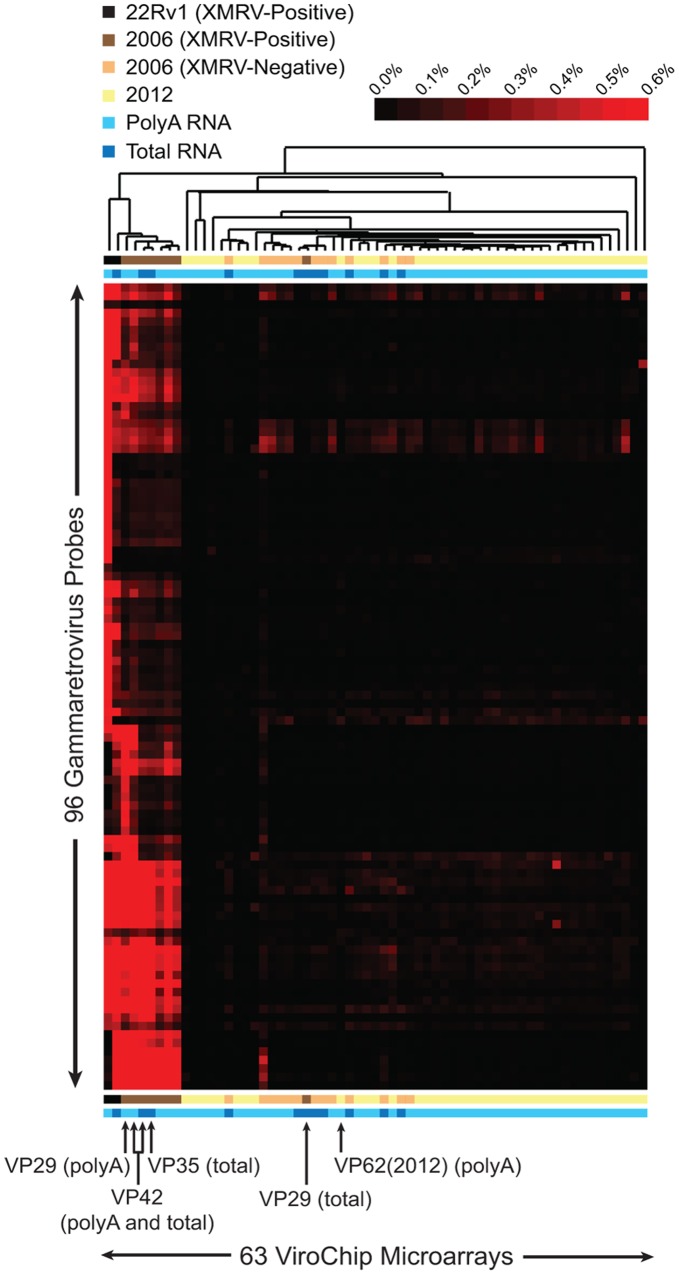
Detection of XMRV in Prostate Cancer Tissues and Archival RNA Extracts by Microarray. Samples were analyzed using the ViroChip, a pan-viral DNA detection microarray (x-axis). The heat map shows a selected cluster consisting of 96 gammaretrovirus probes (y-axis) and corresponding to the same cluster observed in the 2006 study by Urisman, *et al* [1]. The red color saturation indicates the normalized magnitude of hybridization intensity. Microarrays corresponding to key samples are highlighted (arrows). Only prostate cancer samples VP35 and VP42 were found to be consistently positive for XMRV from both total and polyA RNA [1].

The ViroChip microarray [2], [3], [44], [45] was used to screen RNA samples isolated from prostate tumors collected prospectively from 39 individuals, of which 16 individuals were genotyped as harboring the R462Q RNAse L mutation (QQ), 10 individuals were heterozygous cases (RQ), and 13 were wild-type cases (RR). Testing was performed fully blinded so as to remove bias. Archival prostate tissue corresponding to the previously XMRV-positive VP62 sample (QQ) and derived from the same tissue block used in the original 2006 Urisman *et al.* study [1], further referred to as VP62(2012), was also available for microarray screening. Hierarchical clustering with heat map analysis revealed that the 39 tumors as well as the re-extracted VP62(2012) sample were negative for XMRV (Fig. 2, “2012”).

### XMRV Not Detected in Newly Collected Prostate Cancer Samples and Archival VP62 Tissue by Nucleic Acid Testing (NAT)

Three different research institutes (Blood Systems Research Institute, Abbott Laboratories, and Cleveland Clinic) performed independent PCR-based NAT assays for the *gag, pol*, or *env* genes of XMRV, respectively. RNA from prostate cancer tissues corresponding to the 39 individuals identified in the prospective study was initially extracted in a separate mouse-free, XMRV PCR amplicon-free laboratory (University of California, San Francisco). Coded RNA replicates were then distributed in a blinded fashion to each of the 3 laboratories for XMRV NAT. Sensitivity and specificity performance characteristics for each of the 3 NAT assays have been evaluated previously [25], [39], [46]. For all 3 laboratories, none of the 39 samples had detectable XMRV sequences (Table 1). Negative results were also obtained for the archival VP62(2012) sample by all 3 XMRV *gag, pol,* and *env* assays. In contrast, a positive control using GADPH (BSRI or Cleveland Clinic) and β-globulin (Abbott) was successfully amplified for each specimen.

**Table 1:** Detection of XMRV in Prostate Cancer Tissues by PCR.

XMRV Nucleic Acid Testing (NAT) (n = 40)*	QQ (n = 17)*	RQ (n = 10)	RR (n = 13)
NAT-BSRI (XMRV *gag* nested PCR) +	0	0	0
NAT-BSRI (XMRV *gag* nested PCR) −	17*	10	13
NAT-Abbott (XMRV *pol* qRT-PCR) +	0	0	0
NAT-Abbott (XMRV *pol* qRT-PCR) −	16* Δ	10	13
NAT-CC (XMRV *env*) +	0	0	0
NAT-CC (XMRV *env*) −	17*	10	13
GAPDH RT-PCR +	17*	10	13
β-globulin qRT-PCR +	17*	10	13

* includes VP62(2012).

Δ one prospectively collected sample not tested due to lack of sample availability.

### XMRV Detected in RNA Extracts Corresponding to Previously XMRV-Positive Prostate Cancer Samples by Microarray and NAT

A limited number of total or polyadenylated (polyA) RNA samples (n = 21, corresponding to 14 unique samples) from the original 2006 study by Urisman, *et al.* [1], 6 previously found to be XMRV-positive and 8 XMRV-negative, were available for independent re-analysis. The 21 RNA samples were first analyzed using the ViroChip microarray. Consistent with previous results [1], a positive hybridization signal exclusively comprised of gammaretrovirus probes and corresponding to XMRV was detected only in RNA extracts from the 6 previously XMRV-positive samples and the 22Rv1 positive control (Fig. 2; Table 2). After microarray analysis, available remaining material corresponding to 17 total and/or polyA RNA samples (13 unique samples) were tested for XMRV by *gag* RT-PCR. All PCR results were consistent with the microarray data (Table 2), and all of the positive PCR results were sequence confirmed to be XMRV and not another MLV variant, with 98–99% identity to the canonical 22Rv1 XMRV sequence (GenBank accession number FN692043).

**Table 2:** Detection of XMRV in Archival RNA Extracts by Microarray and PCR.

Sample Name	RNAse L Genotype	PreviouslyXMRV(+/−)*	ViroChip(polyA)	ViroChip(total)	RT-PCR(polyA)	RT-PCR(total)	% Identity toXMRV
VP10	QQ	–	–	–	–	NT	
VP27	QQ	–	NT	–	NT	–	
VP29	QQ	+	+	–	NT	–	
VP30	RR	–	–	–	–	–	
VP31	QQ	–	–	–	–	–	
VP35	QQ	+	NT	+	NT	+	99%
VP42	QQ	+	+	+	+	+	99%
VP49	RR	–	–	–	NT	–	
VP50	RR	–	–	–	–	–	
VP51	RR	–	–	NT	NT	NT	
VP79	QQ	+	+	NT	+	NT	99%
VP88	QQ	+	+	NT	+	NT	98–99%
VP90	QQ	+	+	NT	+	NT	98–99%
VP107	QQ	–	NT	–	NT	–	

NT, not tested due to lack of sample availability.

* Urisman, *et al.,* (2006) *PloS Pathogens*, 2(3):e25.

### XMRV Not Detected in Newly Collected Prostate Cancer Samples and Archival VP62 Tissue by FISH

**Figure 3:**
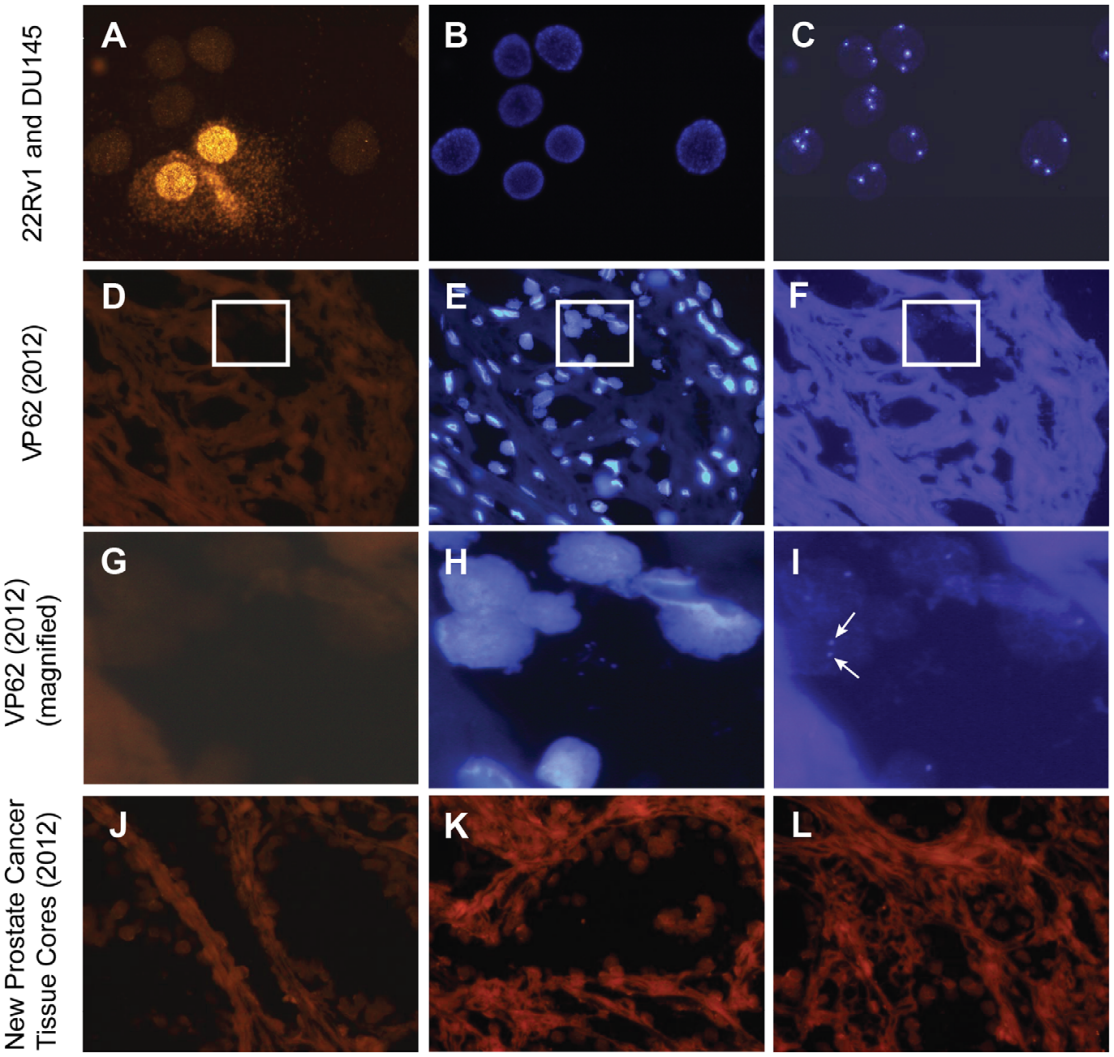
Detection of XMRV in Prostate Cancer Cell Lines and Tissues by FISH. A probe hybridization mix containing XMRV-SO probe (full-length XMRV VP62) and CEP8-SA internal control probe (complementary to a centromeric region of human chromosome 8) was applied to each slide. (**A**) A representative image of XMRV-SO orange staining from a cell mixture of DU145 (uninfected; negative XMRV staining) and 22Rv1 (XMRV-infected; strong positive XMRV staining), showing two positively stained cells. (**B**) DAPI nuclear staining. (**C**) CEP8-SA aqua staining illustrating two and three CEP8 aqua signals per 22Rv1 and DU145 cell, respectively; (**D–F**) Representative images of FFPE prostate cancer tissue sections from patient VP62 (XMRV-SO, DAPI, and CEP8-SA, respectively). No XMRV-SO orange staining is observed. The white rectangle outlines the region magnified in panels G-I (**G–I**) A magnified image of FFPE prostate cancer tissue sections from patient VP62 (XMRV-SO, DAPI, and CEP8-SA, respectively). At this magnification, CEP8-SA aqua staining is clearly visible (panel I; white arrows highlight two representative CEP8 aqua signals). (**J–L**) Representative images showing no XMRV-SO orange staining in FFPE prostate cancer tissue sections from 3 representative patients (among the prospectively collected cohort of 39 patients).

Fluorescence *in situ* hybridization (FISH) was used to screen for the presence of XMRV genomic sequences in formalin-fixed, paraffin-embedded (FFPE) tissue sections from VP62(2012) and 19 newly collected tumors (a subset of the 39 individuals in the prospective study). Among the 19 individuals whose tumors were analyzed, 11 individuals were genotyped as harboring the R462Q RNAse L mutation (QQ), 3 individuals were heterozygous cases (RQ), and 5 were wild-type cases (RR). All specimens were uniformly negative with the XMRV-specific probe (representative fields shown in Fig. 3, D and J-L), although staining from XMRV-infected 22Rv1 cells was positive (Fig. 3A), and a positive internal control signal corresponding to the centromeric region of chromosome 8 (CEP8) was consistently observed (Fig. 3, C, F, and I). Thus, examination of FFPE sections by FISH revealed no evidence of XMRV infection in prostate cancer tissues, regardless of RNAse L genotype.

### No Serological Evidence of XMRV Infection in Prostate Cancer Patients

**Figure 4:**
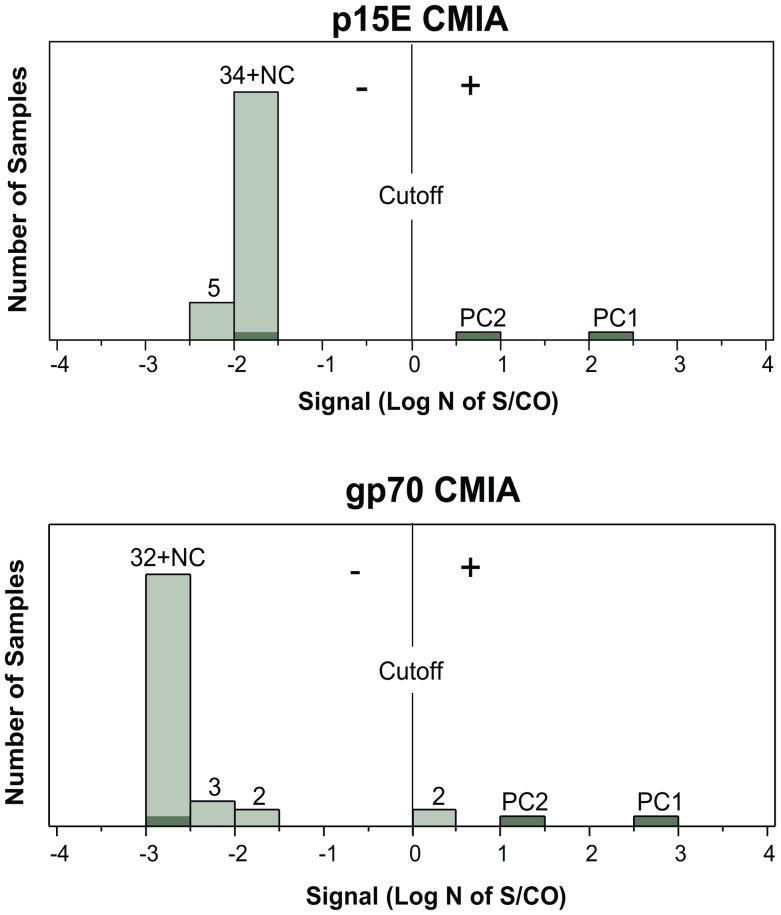
Serological Detection of Antibodies to XMRV in Prostate Cancer Patients. Evaluation of 39 plasma samples from prostate cancer patients for the presence of antibodies to XMRV/MLV using recombinant-based XMRV p15E and gp70 chemiluminsescent microparticle immunoassays (CMIAs) [70]. The x-axis represents the CMIA signal expressed in units of natural log–transformed signal ratio of sample to the cutoff (log N of S/CO); values greater than 0 are considered positive. Signals of the positive controls (PC1 and PC2) corresponding to XMRV-infected macaque plasma and negative control (NC) corresponding to a normal blood donor are highlighted in dark green.

The 39 plasma samples corresponding to the individuals identified in the prospective study were screened for the presence of antibodies to XMRV or other MLVs. None was reactive against the p15E transmembrane protein and only two samples were weakly reactive to the gp70 envelope protein (Fig. 4). However, subsequent testing of the two gp70-reactive samples with a p30 assay revealed no detectable antibodies against the p30 capsid protein. Thus, these data provide no serologic evidence of XMRV or other MLV infection in these patients.

### Full-Length Genomes of XMRV are Present in RNA Extracts from Previously Positive Prostate Cancer Samples

**Figure 5:**
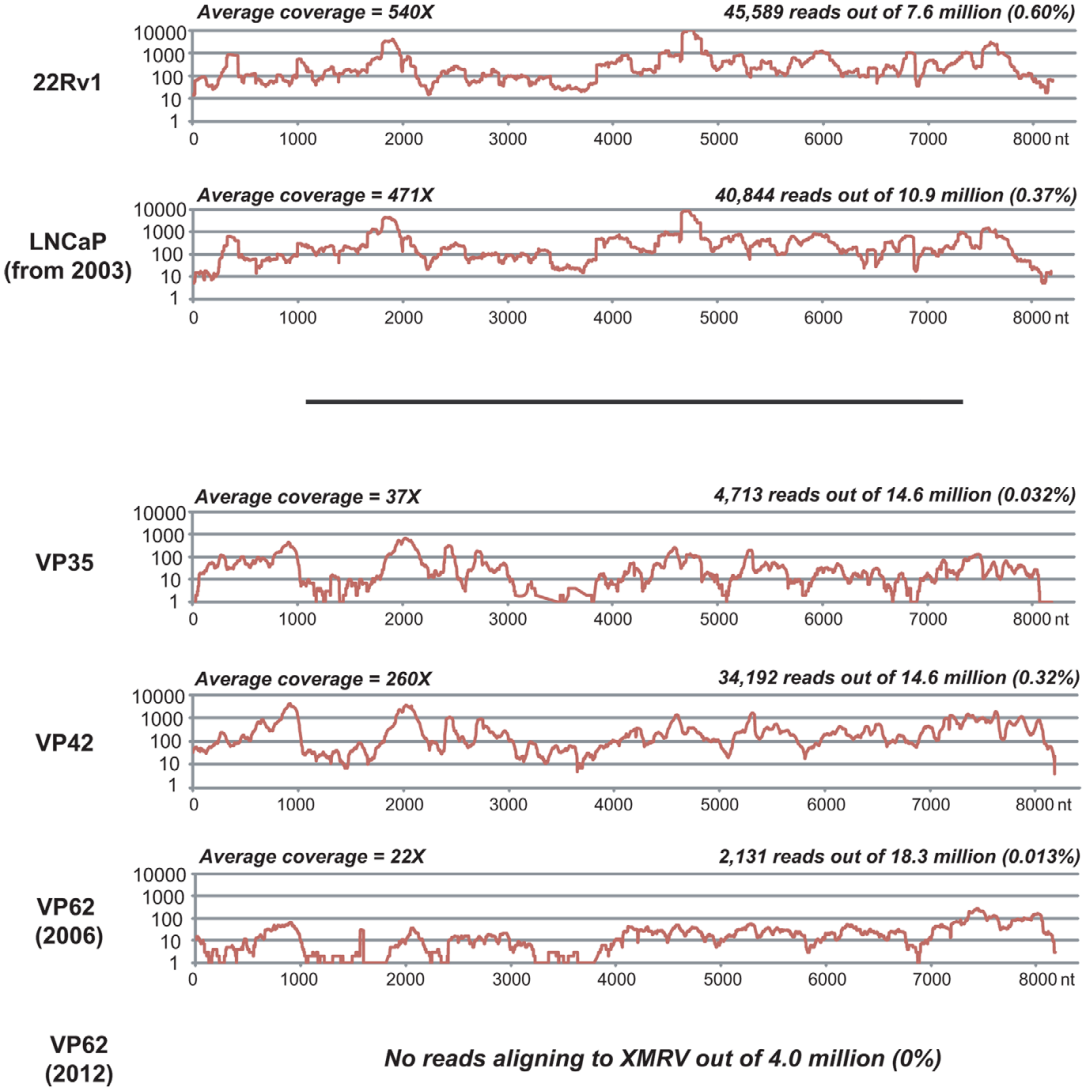
Genomic Coverage of Cell Line and Prostate Cancer-Associated XMRV Strains by Deep Sequencing. RNA extracts of 22Rv1 and LNCaP cells, prostate cancer tissues from the 2006 Urisman, *et al.* study [VP35, VP42, and VP62(2006)], and re-extracted tissue from the VP62 sample, VP62(2012), were analyzed by unbiased deep sequencing. Reads are mapped to the previously sequenced XMRV genome corresponding to each of the samples, with the exception of reads from LNCaP, which are mapped to the 22Rv1-associated genome (Genbank accession number FN692043). The coverage (y-axis) achieved at each position along the ∼8.2 kB XMRV genome (x-axis) is plotted on a logarithmic scale. Abbreviations: nt, nucleotide.

In the original 2006 paper by Urisman, *et al.* [1], three full-length ∼8.2 kb genomes of XMRV were recovered by specific PCR and sequencing of cloned PCR fragments from 3 different prostate cancer samples (VP35, VP42, and VP62). To characterize their viral genomes in greater depth, we analyzed RNA extracts from these 3 samples by unbiased next-generation, or “deep” sequencing. Approximately 14.6 to 18.3 million random shotgun reads were generated per sample, and 4,713, 34,192, and 2,131 reads were mapped to the XMRV genomes corresponding to VP35, VP42, and VP62, respectively (Fig. 5). The mapped reads represented coverage of the entire genome for each of the samples, with the deepest coverage achieved for VP42. *De novo* assembly of >1 kb regions in the absence of a reference genome produced contiguous sequences (contigs) that aligned with highest similarity to XMRV genomes and not to related MLVs (data not shown). No reads corresponding to mouse mitochondrial or intracisternal A-particle (IAP) sequences were detected in these 3 samples by deep sequencing and specific RT-PCR (data not shown). These results suggest that the full-length XMRV genome is present in all 3 clinical samples, and argue against the possibility of mixed infection with other MLV-like retroviruses. Deep sequencing of RNA extracts from the re-extracted archival VP62(2012) tissue was also performed. Importantly, while the RNA from sample VP62 extracted in 2006 was positive for XMRV, RNA extracted from the same prostate tissue in 2012 was found to be negative not only by ViroChip and PCR (Fig. 2; Table 1) but also by deep sequencing, with no XMRV sequences detected out of 4 million deep sequencing reads (Fig. 5).

### Lack of Diversity of XMRV Revealed by Deep Sequencing

**Figure 6:**
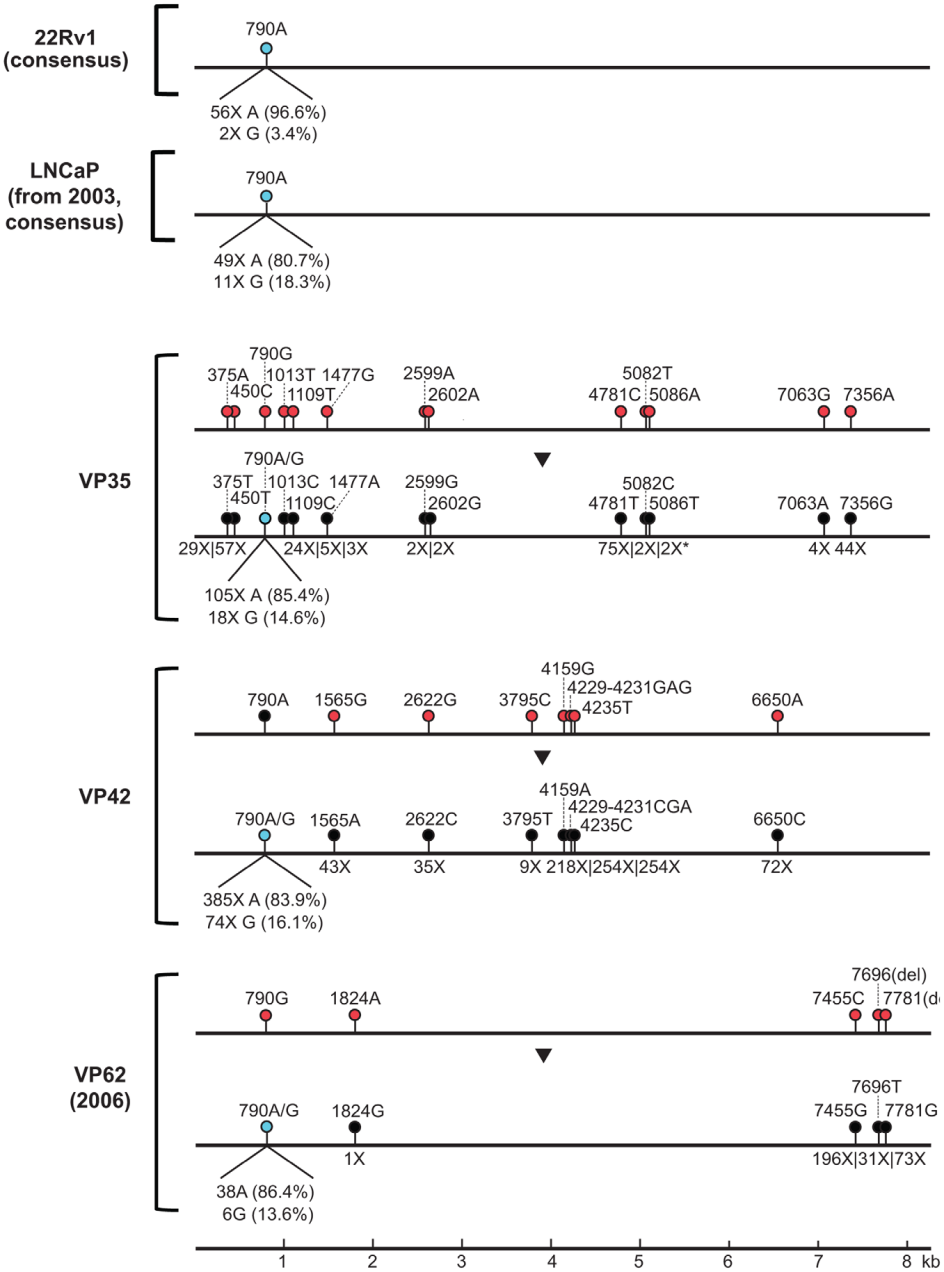
Lack of Diversity Among XMRV Strains Detected in Laboratory Cell Lines and Prostate Cancer Tissues. By SNP analysis, single nucleotide differences between the sequences of 22Rv1-associated XMRV and XMRV genomes detected in prostate cancer tissues [VP35, VP42, and VP62(2006)] (red lollipops) are corrected by the deep sequencing coverage data (black lollipops). The depth of read coverage achieved at the nucleotide position corresponding to each SNP is displayed below the x-axis. All reads covering a given position yielded the same (corrected) nucleotide, indicating that previous nucleotide differences in published genomes [1] (red lollipops) are due to sequencing error. A natural A→G polymorphism in the XMRV genome [23], [38] is present at position 790 (cyan lollipop). Note that XMRV consensus genomes associated with 22Rv1, LNCaP, and the 3 XMRV-positive prostate cancer tissues are identical.

In 2011, Paprotka, *et al.* reported that XMRV likely originated through recombination between 2 endogenous murine retroviruses, PreXMRV-1 and PreXMRV-2, during *in vivo* passaging of the human prostate cancer xenograft CWR-R1, resulting in establishment of the XMRV-infected 22Rv1 cell line [38]. The consensus sequence of 22Rv1-associated XMRV is virtually identical to viral genomes isolated from prostate cancer and CFS patients, including VP35 (13 mismatches), VP42 (8 mismatches), and VP62 (5 mismatches). To determine the precise relationship between the sequences of the consensus 22Rv1 genome and the 3 XMRV genomes recovered in the original 2006 Urisman, *et al.* study [1], a single nucleotide polymorphism (SNP) analysis of the nucleotide differences between the 22Rv1 consensus sequence and the previously published sequences of VP35, VP42, or VP62 was performed (Fig. 6). The SNP analysis reveals that the reported variability in the published genomes from 2006 likely resulted from errors introduced during PCR or sequencing at UCSF and not from inherent phylogenetic diversity of XMRV [47]. When these errors are corrected on the basis of redundant deep sequencing coverage, the final consensus sequences of VP35, VP42, and VP62 are identical to each other and to the 22Rv1 consensus sequence, including a previously described natural polymorphism (A→G) at position 790 [23], [38].

### XMRV Infection of a 2003 LNCaP Cell Line Revealed by Deep Sequencing

The failure to detect XMRV in re-extracted archival material from the VP62 sample and lack of sequence diversity among the XMRV VP35, VP42, and VP62 consensus genomes raised the possibility of contamination of the prostate cancer samples in the 2006 Urisman, *et al.* study by a laboratory-derived cell line, either known to be infected with XMRV, such as 22Rv1 [35], [38], or capable of supporting XMRV infection and potentially infected, such as LNCaP [43], [48]. We reasoned that contamination from XMRV-infected LNCaP cells was more likely given that the laboratory at the Cleveland Clinic performing the nucleic acid extractions in 2004 was simultaneously working with LNCaP and had only worked with 22Rv1 more than 2 years prior. To investigate this possibility, aliquots of 2003 LNCaP cells from the Cleveland Clinic laboratory and 2012 LNCaP cells from the UCSF Cell Culture Facility were tested for XMRV by *gag* RT-PCR (Fig. 1). LNCaP cells handled in the laboratory from 2003 were positive for XMRV, whereas LNCaP cells from 2012 were negative. The XMRV-positive 2003 LNCaP cells were then further analyzed by unbiased deep sequencing. From a total of 10,896,742 raw deep sequencing reads, 40,844 reads were mapped to the 22Rv1-associated XMRV genome (Fig. 5, “LNCaP (from 2003)”), and the resulting consensus assembly was found to be identical to 22Rv1-associated XMRV (Fig. 6, “LNCaP (from 2003, consensus)”).

### Whole-Genome XMRV and Mitochondrial SNP Analysis Supports the Likelihood of Sample Contamination from the XMRV-Infected LNCaP Cell Line

**Figure 7:**
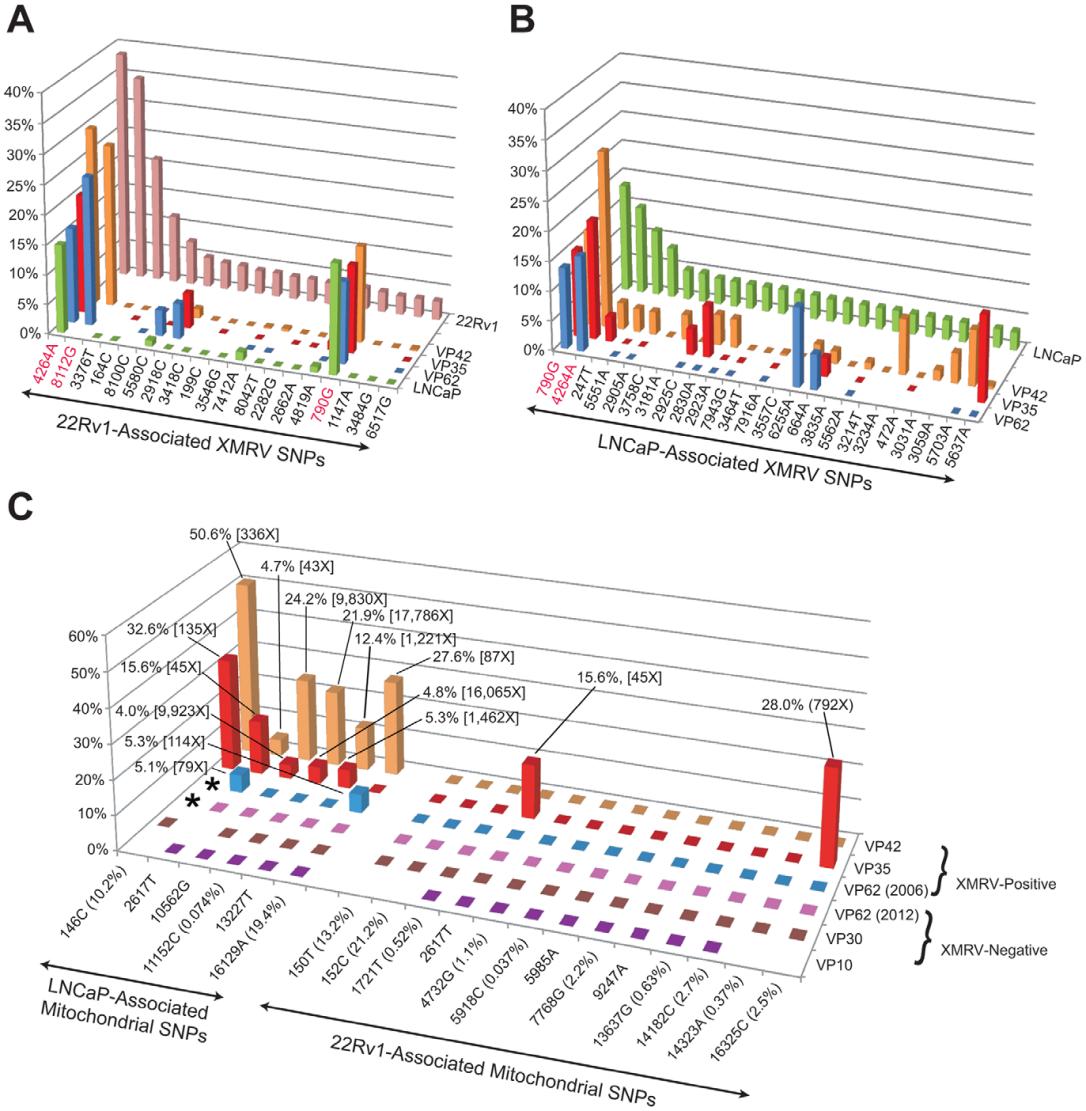
Evidence for Contamination of Prostate Cancer Tissues by XMRV-Infected LNCaP Cells. SNP analysis of deep sequencing reads corresponding to the XMRV genomes of 22Rv1 (**A**) and LNCaP (**B**), as well as the mitochondrial genomes of these two cell lines (**C**) was performed. (**A** and **B**) SNP variants within the XMRV genome for 22Rv1 and LNCaP (x-axis) are shown in order of decreasing frequency (y-axis). Shared SNPs in XMRV genomes corresponding to 3 XMRV-positive prostate cancer samples (VP35, VP42, VP62) and LNCaP (z-axis) at a frequency cutoff of 0.5% are plotted on the graph, with key SNPs highlighted in red. SNPs with variant frequencies <0.5% are plotted as zero; missing values (blank squares) refer to SNP positions for which the coverage is <10X. (**C**) SNP variants within the mitochondrial genome for 22Rv1 and LNCaP are shown (x-axis), with the frequency of each 22Rv-1−/LNCaP-associated SNP in the general human population, as determined by a population-level human mitochondrial database [50], given in parentheses. For each prostate cancer-associated mitochondrial genome (z-axis), the minority SNP frequency (y-axis) is plotted against the cell line-associated SNP variant (x-axis), using a frequency cutoff of 3%. For each minority SNP identified, the variant frequency and coverage at the corresponding nucleotide position is shown. Minority SNPs with variant frequencies <3% are plotted as zero; missing values (blank squares) refer to SNP positions for which the coverage is <30X. Note that the VP62 sample shares a 146C mitochondrial SNP with LNCaP (asterisks).

To characterize the intra-strain diversity of the LNCaP- and 22Rv1-associated XMRV genomes, a SNP analysis of the assembled XMRV genomes at 471X and 540X average coverage, respectively, was performed. Variants within the XMRV genomes were found to be rare, with only 25 SNPs detected in LNCaP and 19 SNPs detected in 22Rv1 at a frequency cutoff of 3% (Fig. 7A and B; Tables S1 and S2). Interestingly, the three most common SNP variants detected in the LNCaP and 22Rv1-associated XMRV genomes, the aforementioned A→G polymorphism at position 790, an A→G polymorphism at position 4264 and a C→G polymorphism at position 8112, can also be found in the VP35, VP42, and VP62 XMRV genomes, with the exception that the C→G polymorphism is not observed in VP35 due to lack of sequence coverage. Overall, more SNPs from LNCaP-associated XMRV than from 22Rv1-associated XMRV were found to be shared with the prostate cancer XMRV genomes. In addition, the variant SNP frequency at the polymorphic 790 position, ranging from 13.6% to 16.4% for the prostate cancer XMRV genomes, was more comparable for the LNCaP-associated XMRV (18.3%) than for the 22Rv1-associated XMRV (3.4%) (Fig. 6). These observations lent additional support for the premise of prostate cancer tissue contamination by XMRV-infected LNCaP, and not 22Rv1, cells.

To further investigate this possibility, we next searched for evidence of direct contamination of prostate cancer samples by XMRV-infected LNCaP or 22Rv1 using a mitochondrial RNA (mtRNA) profiling strategy. The ∼16.5 kb mitochondrial genomes of 22Rv1 and LNCaP were assembled from 555,977 and 171,418 mtRNA deep sequencing reads, respectively. There were 6 nucleotide differences in the LNCaP mitochondrial genome and 13 differences in the 22Rv1 mitochondrial genome relative to the human mitochondrial Cambridge Reference Sequence (CRS) (GenBank NC_012920) [49]. The mitochondrial genomes of VP35, VP42, and VP62 were then assembled from recovered mtRNA reads and scanned for the presence of minority SNPs that would suggest the presence of trace contamination from LNCaP or 22Rv1 cell line-associated mitochondrial sequences (Fig. 7C). Using a cutoff of 3% to define a minority SNP, 6 of 6 SNPs in common between the mitochondrial genomes of VP42 and LNCaP and 5 of 6 SNPs in common between VP35 and LNCaP were detected. Additional scattered minority SNPs were found to be shared among the mitochondrial genomes of VP35, VP42, VP62, LNCaP, and 22Rv1. No SNPs corresponding to either the LNCaP or 22Rv1 mitochondrial genome were found in any of the XMRV-negative samples, including the archival VP62(2012) sample. The presence of additional SNPs that uniquely identify VP35, VP42, or VP62 (data not shown) confirm that prostate tissue RNA was present, and thus, that the shared minority SNPs were the result of LNCaP and/or 22Rv1 contamination. Based on the proportions of SNP variants in the human population as estimated from a population-level mitochondrial database (mtDB) [50], the probability of sharing all 6 SNPs in common between VP42 and LNCaP by random chance alone is estimated at less than 0.00146%.

## Discussion

In this study, we investigated the putative association between XMRV and prostate cancer using a combination of microarray, PCR, FISH, serological, and deep sequencing approaches. XMRV was not detected in a new set of 39 prospectively collected prostate tumors (both with or without RNAse L mutations) by PCR assays performed independently in 3 different laboratories or ViroChip microarray. Moreover, XMRV was not detected in archival VP62 tissue previously found to be XMRV-positive [1]. These negative findings were supported by the failure to detect XMRV sequences in 19 of the newly collected prostate cancer samples and archival VP62 tissue by FISH. In addition, we failed to detect antibody responses to XMRV in plasma samples from the 39 patients with prostate cancer, a finding that was also observed recently in another study [51] Taken together, the data presented here strongly suggest that there is no association between XMRV infection and prostate cancer, regardless of RNAse L status.

In the original 2006 study by Urisman, et al. as well as a 2010 study by Arnold, et al. [1], [9], XMRV was detected in a small proportion of nonmalignant stromal cells by FISH. It is unclear as to why XMRV was detected by FISH in these previous studies but not in the current study, which included re-analysis of archival VP62 tissue. Here we used a direct-labeled, full-length plasmid XMRV probe with a high label incorporation rate [52], which produced a clear punctate staining pattern in 22Rv1 cells by FISH (Fig. 3A). Of note, this novel probe design was able to detect human papillomavirus (HPV) in cervical cancer cells harboring 1 to 2 copies of integrated HPV-16 per cell as well as in cervical cancer tissue sections (data not shown). Thus, if integrated XMRV was present in the tissues examined in this study, it should have been detected by FISH. It is likely that the low frequency of XMRV FISH-positive prostate cells observed in previous studies [1], [9] represent non-specific binding artifacts.

To investigate whether the discovery of XMRV may have resulted from inadvertent laboratory contamination, we re-analyzed available archival RNA extracts from prostate cancer samples taken from the original 2006 study by Urisman, *et al* [1]. By microarray and PCR analysis, the previous findings that a subset of these samples harbored XMRV sequences was replicated (Fig. 2; Table 2). Furthermore, unbiased deep sequencing analysis of 3 XMRV-positive samples (VP35, VP42, and VP62), revealed that the entire viral genome was present (Fig. 5). Failure to detect mouse mitochondrial or IAP sequences in these 3 samples also support the contention that these samples harbor XMRV and not related mouse endogenous gammaretroviruses.

One of the findings arguing against laboratory contamination as a possible source of XMRV has been the degree of sequence variation observed between XMRV genomes, up to 2% in the *gag* and *pol* fragments [1], [14]. Although the reported diversity is extremely low for retroviruses in general [53], certain retroviruses such as HTLV-1 can exhibit comparably low rates of natural sequence variation, with strains in the wild that are 96–99% identical [54]. Nevertheless, the SNP data generated from deep sequencing reveal that the consensus sequences of the XMRV VP35, VP42, and VP62 genomes are in fact identical to each other and to the consensus 22Rv1-associated XMRV strain (Fig. 6). Thus, previously reported sequence diversity between different strains in the 2006 study by Urisman, *et al.* [1] and presumably in other fully- or partially-sequenced XMRV genomes appears to arise from Taq polymerase errors introduced during PCR and/or sequencing [47], and not from natural genetic variation.

Notably, we found evidence of XMRV infection of a 2003 LNCaP prostate cancer cell line by deep sequencing (Fig. 5). The consensus sequence of the XMRV genome in these LNCaP cells was found to be identical to the 22Rv1 XMRV consensus sequence. Both of these cell line-associated XMRV genomes were found to exhibit a lower degree of intra-strain variation than previously reported for XMRV from 22Rv1 cells [20], with only 19 SNPs detected in the 22Rv1-associated XMRV genome at the 3% frequency cutoff by deep sequencing, and only 25 SNPs in the LNCaP-associated genome (Fig. 7A; Table S1). It is therefore striking that the three most common SNP variants identified in LNCaP- and 22Rv1-associated XMRV by deep sequencing, A790G, A4264G, and C8122G, are also present in the 3 prostate cancer-associated XMRV genomes. In conjunction with the 100% consensus sequence identity shared among cell line and prostate cancer-associated XMRV genomes (Fig. 6), these findings suggest a high likelihood that a viral contamination event had occurred.

To prove the hypothesis that an XMRV-infected cell line had contaminated the prostate cancer samples in the 2006 Urisman, *et al.* study, we analyzed available RNA extracts using a novel technique referred to as mitochondrial RNA (mtRNA) profiling. Unlike profiling strategies involving whole or partial genome sequencing of mitochondrial DNA [55], [56], here the ∼16.5 kb mitochondrial genome is assembled from only RNA-derived deep sequencing reads. By mitochondrial SNP analysis, direct evidence of contamination from LNCaP mitochondrial sequences in the VP35 and VP42 samples was detected, with surprisingly high minority SNP frequencies ranging from 4.0% to 50.6% (Fig. 7C). Importantly, VP35 and VP42 were among the first 10 samples processed in the original 2006 study by Urisman, *et al.* [1], and the only two samples positive for XMRV from both total and polyA RNA (Fig. 2, Table 2, and [1]). Taken together, these findings imply that the initial contamination event, involving VP35 and/or VP42, occurred very early in the course of the 2006 study.

**Figure 8:**
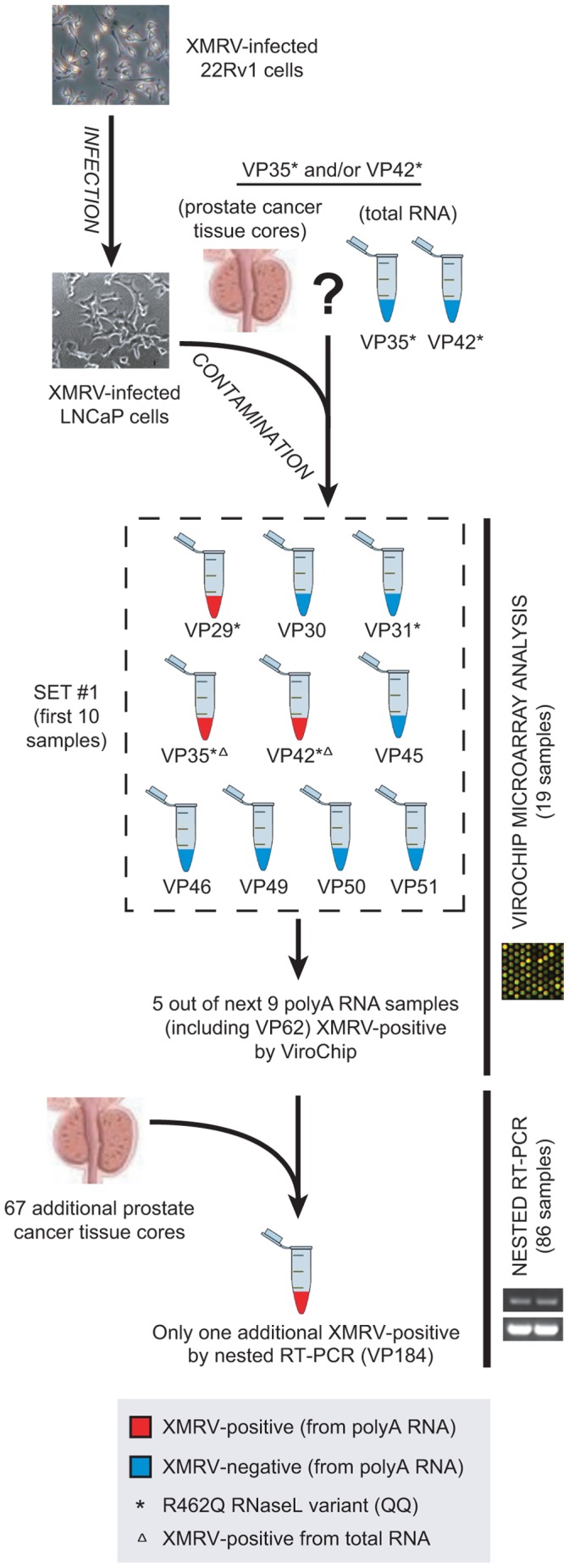
Proposed Model for Laboratory Contamination by XMRV. Early contamination of VP35/VP42 prostate cancer tissues and/or extracted RNA by XMRV-infected LNCaP cells resulted in mistaken identification of XMRV in association with prostate cancer.

In light of the data presented here, we have generated a model for how XMRV contamination was introduced into the prostate cancer samples analyzed by Urisman, *et al.* [1] (Fig. 8). An XMRV-infected LNCaP cell line in the laboratory at the Cleveland Clinic inadvertently contaminated RNA from VP35 and VP42 during RNA extractions, which comprised part of the initial set of 10 samples that were extracted on the same day (Fig. 8, “SET #1″). The LNCaP cell line, in turn, had been likely infected with XMRV from 22Rv1 cells in the same laboratory in which 22Rv1 cells were previously used, or in another laboratory at the Cleveland Clinic that was working with both cell lines and had initially provided the LNCaP cells for analysis. It should be emphasized, however, that only after 2009 was XMRV known to be present in 22Rv1 cells or in any other cell line [35]. In fact, as a necessary precaution, all cell lines circulating in the laboratory were tested in 2004 for XMRV and all tested negative by RT-PCR with the exception of a different but related MLV from an aliquot of LNCaP whose genome at the time was fully sequenced (“MLV-LNCaP”). Based on this analysis, it was mistakenly deduced that XMRV could not have originated from LNCaP or another cell line in the laboratory. Interestingly, in the current study, we were unable to recover sequence from this related MLV in a fresh aliquot of 2003 LnCaP cells by deep sequencing, and instead, found only XMRV (Fig. 5; Fig. S1). After contamination of the VP35 and/or VP42 sample(s) by XMRV-infected LNCaP, polyA RNA extracts from other prostate cancer samples then became cross-contaminated. The detected association of XMRV with the RNAse L R462Q variant (QQ) may have resulted in part from an increased proportion of QQ samples analyzed (11 of 19; 58%) relative to the QQ genotype frequency in prostate cancer cases of approximately 15% [5]. Notably, after detection of 8 XMRV-positive samples (out of 19) by ViroChip and PCR, subsequent PCR screening of an additional 67 prostate cancers yielded only one additional positive sample, VP184 [1], which in hindsight may represent nested PCR contamination.

In summary, our findings do not support any association between XMRV infection and prostate cancer, and by extension indicate that XMRV has never replicated outside of the laboratory setting. The initial discovery linking XMRV to prostate cancer in 2006 arose from laboratory contamination of clinical samples by an XMRV-infected LNCaP cell line. In turn, the LNCaP cells were most likely previously infected by 22Rv1, from which XMRV almost certainly originated through *in vivo* passaging of the CWR22 xenograft in mice [38]. Nevertheless, the discovery of XMRV in 2006 accelerated research that has now established the virus as a genuine infectious agent with a unique biology and as-yet undefined pathogenic potential. Important features of XMRV biology include (1) tropism for a variety of cell lines, including prostate cancer DU145 and LNCaP cells [27], [43], [48], and human neural cell types [57], (2) adaptations that promote growth in prostate epithelium and human-derived prostate cancer cell lines including an androgen response element in the promoter region [58] and downregulation of APOBEC3G [59], and (3) cellular effects with potential oncogenic properties including increased tumor aggressiveness mediated by downregulation of p27 [60] and differential regulation of several microRNAs [61]. A study of XMRV-induced apoptosis of SY5Y human neuroblastoma cells identified its receptor, Xpr1, as a novel atypical G-protein-coupled receptor (GPCR) [62]. Finally, XMRV was found to establish both acute and chronic infections in mice and two species of non-human primates [23], [63], [64].

## Methods

### Ethics Statement

Human specimens, including prostate tissue and blood plasma, were collected from prostate cancer patients for analysis under protocols approved by the Cleveland Clinic Foundation Institutional Review Board (IRB). Written informed consent was obtained for all patients.

### Clinical Samples

Plasma was prepared from blood collected in either EDTA or sodium citrate vacutainer tubes and stored at −70°C. Fresh frozen prostate tissues were collected and stored as described previously [1]. Briefly, prostate tissue cores were collected immediately after prostatectomies, frozen in liquid nitrogen and then stored at −80°C. Remaining prostate tissue was formalin-fixed and paraffin-embedded (FFPE), sectioned with a microtome and placed on microscope slides for FISH analysis (referred to as FFPE sections).

### Nucleic Acid Extraction

Forty prostate core tissues were transported directly from the tissue pathology repository at the Cleveland Clinic and were extracted at University of California, San Francisco (UCSF) using TRIzol (Invitrogen). Tissues were minced using single-use, sterile scalpels alternated with 3 freeze-thaw cycles and extracted according to the manufacturer’s protocol. Linear acrylamide (20 µl) was used as an RNA carrier during isopropanol precipitation. Subsequent RNA was treated using either DNAse (Zymogen RNA Cleanup; Ambion Turbo DNase) or polyA-selection (Qiagen Oligotex) according to the manufacturer’s instructions. RNA from cell cultures of 22Rv1 and LNCaP were extracted similarly using TRIzol LS (Invitrogen).

### ViroChip Microarray Analysis

ViroChip analysis was performed as previously described [2], [3], [44], [45]. Briefly, RNA was reverse-transcribed to cDNA using random primers (5′-(N_9_)GTTCCCACTGGAGGATA-3′) and second-strand synthesis was performed using Sequenase. Samples were labeled with Cy3 fluorescent dye, normalized to 10 pmol of incorporated dye, and hybridized overnight to the ViroChip microarray for 16 hr at 65°C. The current 8×60 k version 5.0 (v5.0) ViroChip microarrays used in this study (GEO accession number GPL11662) are manufactured commercially on an Agilent platform (Agilent Technologies), and contain 19,058 70 mer oligonucleotide probes representing all viral species in GenBank. These viral probes encompass probes from all previous ViroChip designs, including the v2.0 design used in the 2006 study by Urisman, *et al.* (GEO accession number GPL1834). Microarrays were scanned at 2 µm resolution on an Agilent DNA Microarray Scanner. Sum normalization of microarrays was performed using the background-subtracted intensities corresponding to the 4,672 viral probes in common between the v2.0 and v5.0 ViroChip designs. Microarray hybridization patterns were interpreted using hierarchical cluster analysis as previously described [65], [66]. Specifically, for the cluster (heat-map) analysis, normalized intensities of all retroviral probes in common between the two designs (n = 492) were used to cluster microarrays and probes. The sub-cluster of 96 gammaretrovirus probes displayed in Fig. 2 is the same cluster as previously observed in the 2006 study by Urisman, *et al.* [1]. All ViroChip microarrays used in this study have been deposited in the NCBI GEO database (accession numbers GSE39684 and GSM977688-GSM977750).

### XMRV *gag* nested RT-PCR (Blood Systems Research Institute)

For detection of XMRV *gag* sequences by nested RT-PCR, ∼200 ng of extracted RNA were first subjected to reverse transcription using the Superscript III First-Strand Synthesis System for RT-PCR (Invitrogen) according to the manufacturer’s instructions. Next, 5 µl of the transcribed cDNA were used for the first round of PCR amplification with primers 419F (5′-ATCAGTTAACCTACCCGAGTCGGAC-3′) and 1154R (5′-GCCGCCTCTTCTTCATTGTTCTC-3′) [14] and HotStart-IT FideliTaq Master Mix (USB) with the recommended component volumes. The template was denatured for 4 min at 94°C and amplified in 40 cycles of 1 min at 94°C, 1 min at 57°C and 1 min at 72°C, followed by a final incubation for 10 min at 72°C. Nested PCR was performed under the same conditions for 45 amplification cycles with 5 µl of the first round PCR product and two different primer pairs, Gag-I-F (5′-TCTCGAGATCATGGGACAGA-3′) and Gag-I-R (5′-AGAGGGTAAGGGCAGGGTAA-3′) or NP116 (5′-CATGGGACAGACCGTAACTACC-3′) and NP117 (5′-GCAGATCGGGACGGAGGTTG-3′), respectively, both of which have been shown to detect both XMRV and MLV *gag* sequences [13]. Serial dilutions of a cloned fragment of XMRV *gag* [28] ranging from 1 to 100 copies/µl were included in each PCR to determine the assay sensitivity, which was found to be ∼1 copy/µl. The resulting PCR amplification products were analyzed by electrophoresis in 1.5% agarose gels. Bands of approximately the correct size were excised and subjected to sequencing to determine potential homology to XMRV or MLV.

### XMRV *pol* RT-PCR (Abbott Molecular, Inc.)

Total RNA extracted from prostate cancer tissue cores was tested in the single-round XMRV *pol* RT-PCR assay utilizing the *m*2000rt system (Abbott Molecular, Inc.; Desplaines, IL). An average of ∼500 ng of total RNA input per reaction was diluted in water to a total volume of 25 µl, and 25 µl of master mix that contained EZ buffer, rTth enzyme, dNTPs, Rox reference dye, MnCl2 and primer/probe was added to obtain a final reaction volume of 50 µl. A primer/probe set designed to detect 136 bases of the human β-globulin gene was amplified and detected simultaneously in the same reaction with XMRV *pol* sequence to control for specimen integrity and PCR efficiency. The details related to primer/probe sequences, cycling conditions and sensitivity/specificity estimation of the *pol* RT-PCR have been described previously [46].

### XMRV *env* qRT-PCR (Cleveland Clinic)

Coded RNA samples from prostate cancer tissue biopsies were shipped from UCSF to the Cleveland Clinic. Samples were transferred to an XMRV amplicon-free clean room and diluted to 100 ng/µl with nuclease free water (USB/Affymetrix), 50 ng of which was used per assay. The AgPath-ID One-Step qRT-PCR Kit (Ambion) was used for the qRT-PCR assay with the *env* primers/probe combination (6124F, 6159R and 6197R) [39] according to the manufacturer’s protocol. Assays were performed on a StepOnePlus Real-Time PCR System (Applied Biosystems). Reactions (25 µl) were heated to 45°C for 10 min followed by 95°C for 10 min. The reactions were then subjected to 55 cycles at 95°C for 15 sec and 60°C for 1 min. For the GAPDH RT-PCR assay, 1.25 µl of TaqMan pre-developed human GAPDH primers (Applied Biosystems) was used in a final reaction volume of 25 µl. PCR conditions were the same except that the cycle number was reduced to 45 as GADPH RNA was present at relatively high copy numbers. For each assay, a standard curve was generated with known copy numbers of XMRV RNA.

### Preparation of Human Prostate Cancer Cell Lines DU145 and 22Rv1 for FISH

DU145 cells (XMRV-uninfected) [39], and 22Rv1 cells carrying at least 10 integrated copies of XMRV [31], were used as a negative and positive control, respectively, in XMRV FISH assays. These cells were propagated in DMEM-F12 complete medium (Invitrogen) at 37°C in an atmosphere of 5% carbon dioxide. After growing to a 60% to 70% confluence level, cells were arrested with 1 ml of Colcemid solution (10 µg/ml) (Invitrogen) for every 50 ml cell culture and incubated at 37°C for 2 hours. Cells were then harvested using a standard trypsinization procedure. After washing collected cells once with 40 ml of 1×DPBS (Invitrogen), cells were resuspended in 40 ml of 0.075 M potassium chloride solution (Invitrogen) and incubated at 37 °C for 30 min. Cells were subsequently washed 4 times each with 40 ml of Carnoy’s fixative (methanol:glacial acetic acid = 3∶1 (v/v)) (Fisher, Pittsburgh, PA), then resuspended in 5 ml of Carnoy’s fixative and stored at −20°C. Slides having a mixture of DU145 and 22Rv1 were prepared by depositing cells (10 µl per slide for each of the two cell suspensions) on SuperFrost Plus positively charged slides (ThermoShandon, Pittsburgh, PA). Cell-coated slides were then air-dried overnight prior to pretreatment and hybridization.

### Generation of FISH Probes

DNA of a plasmid clone VP62/pcDNA3.1 having a full-length (∼8.2 kb) XMRV VP62 genome [39] was extracted using PureLink MaxiPrep DNA Kit (Invitrogen) according to the manufacturer’s instructions. The entire plasmid DNA (∼13.6 kb) was then directly labeled with SpectrumOrange fluorophore to generate XMRV-SO FISH probe as described previously [47]. The percentage of SpectrumOrange incorporation was approximately 8%. CEP8-SA probe complementary to human chromosome 8-specific centromeric repetitive sequences and directly labeled with SpectrumAqua fluorophore was obtained from Abbott Molecular, Inc. (Des Plaines, IL).

### Specimen Pretreatment and *in situ* Hybridization for FISH

Slides containing a mixture of DU145 and 22Rv1 cells (see Figure S1, Table S1, Table S2) were pretreated in 2×SSC (0.3 M NaCl, 0.03 M sodium citrate, pH 7.0 (Invitrogen)) at 73°C for 2 min, followed by a 10-min incubation in pepsin solution (0.5 mg/ml pepsin in 10 mM HCl; USB, Cleveland, OH) at 37°C. Slides at room temperature were then rinsed in 1×DPBS (Invitrogen) for 5 min, fixed in a 1% neutral-buffered formalin solution (Fisher) for 5 min and rinsed again in 1×DPBS for another 5 min. Dehydration of slides was performed through a series of ethanol: 1 min each in 70%, 85%, and 100%, followed by air-drying. A 10 µl of probe hybridization mix was prepared by mixing 100 ng XMRV-SO, 100 ng CEP8-SA, 1000 ng sonicated human placental DNA, 250 ng human Cot-1 DNA, and 7 µl LSI/WCP hybridization buffer (Abbott Molecular, Inc.), and was added to each slide over the cell specimen. Coverslips (22×22 mm; VWR, Radnor, PA) were placed on the slides and sealed with rubber cement (Staples, Framingham, MA). Probes and cell nucleic acids on each slide were co-denatured for 3 min at 73°C and immediately hybridized for 16–24 hours at 37°C on a ThermoBrite (Abbott Molecular, Inc.). Following hybridization, coverslips were removed, and slides were washed in 0.4×SSC/0.3% NP-40 (Abbott Molecular, Inc.) at 73°C for 2 min and then in 2×SSC/0.1% NP-40 (Abbott Molecular, Inc.) for 1 min at room temperature. Ten µl of DAPI II (125 ng/ml; Abbott Molecular, Inc.) counterstain was placed on each slide, and a coverslip was applied.

All slides mounted with FFPE human prostate cancer tissue sections were baked at 56°C for 4 hours to fix the tissue onto the slides and were then stored at room temperature. In preparation for *in situ* hybridization, tissue specimen slides at room temperature were deparaffinized by soaking in three changes of Hemo-De solvent (Scientific Safety Solvents, Keller, TX) for 5 min each, followed by two 1-minute rinses in 100% ethanol, an incubation in a solution of 45% formic acid (Fisher)/0.3% hydrogen peroxide (Calbiochem) for 15 min, and a rinse in water for 3 min. Slides were then immersed in pretreatment solution (Abbott Molecular, Inc.) at 80°C for 35 min, rinsed for 3 min in water at room temperature, incubated for 22 min in pepsin solution (1.5 mg/ml in 0.1 N HCl) at 37°C, and rinsed again for 3 min in water at room temperature. Slides were subjected to dehydration for 1 min each in 70%, 85%, and 100% ethanol, and were then air-dried. A 10 µl of probe hybridization mix was made by mixing 100 ng XMRV-SO, 100 ng CEP8-SA, 1000 ng sonicated human placental DNA, 250 ng human Cot-1 DNA, and 7 µl LSI/WCP hybridization buffer, and was dropped to each slide over the tissue section. Slides were then coverslipped and sealed with rubber cement. Probes and specimen nucleic acids on each slide were co-denatured for 5 min at 73°C and immediately hybridized for 16–24 hours at 37°C on a ThermoBrite. Following hybridization, slides were soaked in 2×SSC/0.1% NP-40 at room temperature for 5–10 min for coverslips to come off, then washed in 0.4×SSC/0.3% NP-40 at 73 °C for 2 min, and subsequently in 2×SSC/0.1% NP-40 for 1 min at room temperature. Ten µl of DAPI I (1,000 ng/ml; Abbott Molecular, Inc.) counterstain was added over each tissue section, and a coverslip was applied. After adding nuclear counterstain DAPI, slides were examined under a fluorescence microscope. XMRV-SO orange staining, CEP8-SA aqua staining and DAPI nuclear staining were visualized, respectively, with filters of orange, aqua and DAPI (Abbott Molecular, Inc.).

### Chemiluminescent Microparticle Immunoassay (CMIA) Testing for XMRV

A detailed procedure has been described previously [21], [67], [68], [69]. Briefly, 100 µl of plasma were screened in two direct format ARCHITECT chemiluminescent immunoassays (CMIAs; Abbott Diagnostics, Abbott Park, IL) that utilize recombinant XMRV p15E or gp70 protein [70]. These assays have previously been shown to be both specific (99.5%–99.9%) and highly sensitive (100%) based on studies performed in rhesus macaques and human blood donors [70]. Assay positive controls (PCs) were derived from XMRV-infected rhesus macaque plasma at 1∶1000 (PC1) or 1∶4000 (PC2). A pool of normal human plasma was used as a negative control (NC). Any sample that gave a signal equal to or greater than the cutoff value (Log N S/CO≥0.0) was repeated in duplicate, and samples with repeatedly reactive results were further analyzed by the ARCHITECT XMRV p30 CMIA for antibodies to capsid protein. Repeat reactivity (Log N S/CO≥0.0) to all three proteins is required to confirm a positive antibody finding [67], [68], [71].

### Library Preparation for Deep Sequencing

For the XMRV-positive and XMRV-negative prostate cancer samples, randomly amplified cDNA was prepared for deep sequencing using a variation of Illumina’s TruSeq protocol (Illumina) as previously described [2], [45]. Briefly, amplified cDNA samples were cleaned using AMPure SPRI beads (Agencourt AMPure XP) and digested using the restriction enzyme *BpmI* (New England Biolabs, Ipswich, MA) for 2 hr at 37°C, followed by end-repair and A-tailing with Klenow and Taq polymerase, respectively (Invitrogen). Size selection for ∼250 base pair (bp) fragments was then performed using AMPure beads, and sequencing adaptors containing 6-nucleotide barcode tags were attached according to an Illumina paired-end protocol. For the 22Rv1 and LNCaP cell lines, the Illumina ScriptSeq v.2 kit (Illumina) was used for library generation according to the manufacturer’s instructions.

Final libraries were analyzed using the Bioanalyzer DNA 12000 chip for assessing size distribution (Agilent) and SYBR©FAST qPCR for confirming properly adapted DNA fragments (KAPA Biosystems). Either 100 bp or 150 bp paired-end resequencing was then performed using Illumina HiSeq or MiSeq instruments, respectively. Deep sequencing reads were submitted to the NCBI Sequence Read Archive (accession number SRA056286).

### Mapping of Deep Sequencing Reads to XMRV Genomes

Raw sequence reads (both single reads and their mate pairs) corresponding to the VP35, VP42, VP62(2006), VP62(2012), 22Rv1, and LNCaP deep sequencing libraries were stripped of adapter and primer sequences and aligned to a custom database of all gammaretrovirus sequences in GenBank using BLASTn (word size = 11, E-value = 1×10^−10^) [72]. Hits with a better match in the GenBank nonredundant nucleotide database (NT), corresponding predominantly to human genomic background sequences resulting from misannotations in GenBank, were excluded from the analysis. Mapping of remaining gammaretrovirus reads to the designated XMRV reference genomes or *de novo* assembly was then performed using Geneious software [73]. Specifically, for the XMRV SNP analysis, reads were initially trimmed for quality by trimming 6 bp from the 5′ and 3′ ends, trimming regions with more than a 0.1% chance of an error per base, removing all low-quality bases, and setting the number of maximum ambiguities to 1. These high-quality reads were then mapped to the corresponding XMRV reference genome using the following parameters (no gaps allowed, maximum mismatches allowed per read of 5%, and maximum ambiguity of 1). A consensus sequence based on mapped deep sequencing reads was generated for each of the prostate cancer XMRV genomes and used to correct errors in the previously published sequences, with the requirement of no ambiguity at each discrepant nucleotide position.

### Screening for Contaminating Mouse Sequences by Deep Sequencing and PCR

To search for mouse genomic contamination, sequencing reads corresponding to VP35, VP42, and VP62(2012) were aligned to the mouse-specific mitochondrial cytochrome b gene (GenBank accession number NC_005089.1; nucleotide positions 14154–15246) using BLASTn (word size = 11, E-value = 1×10^−10^) [72]. An RT-PCR assay for the detection of mouse IAP sequences was also performed on total RNA extracts from VP35 and VP42 and polyA RNA extracts from VP62(2006) (as no total RNA was available) using previously published primers and conditions [42].

### XMRV and Mitochondrial Single Nucleotide Polymorphism (SNP) Analysis

SNP variants in the 22Rv1 and LNCaP-associated XMRV genomes were called in Geneious using a minimum variant frequency cutoff of 3% and minimum coverage of 30. Following identification of SNP variants, the corresponding nucleotide positions in mapped XMRV VP35, VP42, and VP62 genomes were scanned for the presence of shared SNPs using a minimum variant frequency cutoff of 0.5% and minimum coverage of 10. These less-stringent threshold parameters were chosen because of the more shallow depth of coverage for the prostate cancer-associated XMRV genomes.

For the mitochondrial SNP analysis, the mitochondrial genomes of 22Rv1 and LNCaP were first assembled using the Cambridge Reference Sequence (CRS) human mitochondrial genome (GenBank NC_012920) [49] as a scaffold. Mitochondrial reads were identified using BLASTn (word size = 11, E-value = 1×10^−10^) [72] and mapped in Geneious [73]. In total, 554,762 high-quality mtRNA sequences out of the 7,567,228 raw deep sequencing reads generated from the 22Rv1 cDNA library were mapped to the CRS mitochondrial genome, and a consensus sequence of the ∼16.6 bp 22Rv1 mitochondrial genome was generated with a mean coverage of 3,172X. For the LNCaP deep sequencing library, 171,418 reads out of 10,896,742 were mapped to the CRS mitochondrial genome, producing a consensus sequence with a mean coverage of 955X. Raw single reads (and their mate pairs) from deep sequencing libraries corresponding to 3 XMRV-positive samples [VP35,14,589,296 reads; VP42, 14,573,990 reads; and VP62(2006), 18,308,352 reads] and 3 XMRV-negative samples [VP10, 5,270,536 reads; VP30, 4,378,204 reads; and VP62(2012), 3,985,692 reads] were then stripped of adapter and primer sequences and aligned to the CRS mitochondrial genome using BLASTn (word size = 11, E-value = 1×10^−10^). Reads were trimmed in Geneious and mapped to the CRS mitochondrial genome using the same parameters as used for the XMRV genome mapping. Mapped reads were then examined for the presence of minority SNPs corresponding to any of the 19 or 25 SNPs identified in the 22Rv1 or LNCaP mitochondrial genomes, respectively. For a variation to be called a minority SNP, the nucleotide change had to be identical to the cell line-associated SNP, with a minimum coverage of 30 and minimum variant frequency of 3% at that position. Approximate p-values were calculated in Geneious [73] assuming a minimum base quality of 20, or that the reads are >99.0% correct (Tables S1 and S2). The proportion of each LNCaP−/22Rv1-associated SNP variant in the general human population, with the exception of SNPs 2617T, 10562G, 13227T, 2617T, 5985A, and 9247A for which information was not available, was estimated by searching mtDB, a population-level database of sequenced human mitochondrial genomes [50].

## Supporting Information

Figure S1 **Assembly of Deep Sequencing Reads from XMRV-Infected LNCaP Cells to XMRV and MLV-LNCaP.** In 2004, the genome of an MLV related to XMRV (“MLV-LNCaP”) was sequenced from LNCaP cells. In the current study, deep sequencing reads generated from an XMRV-infected 2003 LNCaP cell line were mapped to the genomes of canonical 22Rv1-associated XMRV (GenBank accession number FN692043) and MLV-LNCaP. The 100% identity shared between the consensus XMRV genomes of 2003 LNCaP and 22Rv1 (**A**), and significant discrepancies between the consensus XMRV genome of 2003 LNCaP and MLV-LNCaP (**B**) indicate that 22Rv1-associated XMRV, and not MLV-LNCaP, is present in the 2003 LNCaP cells.
(PDF)

Table S1 **SNPs in the 22Rv1-Associated XMRV Genome and Comparison to the Prostate Cancer and LNCaP-Associated XMRV Genomes.** Approximate p-values are calculated assuming a minimum base quality of 20, or that the reads are >99.0% correct.
(PDF)

Table S2 **SNPs in the LNCaP-Associated XMRV Genome and Comparison to the Prostate Cancer and 22Rv1-Associated XMRV Genomes.** Approximate p-values are calculated assuming a minimum base quality of 20, or that the reads are >99.0% correct.
(PDF)
