# Supplementary figures and images for: In-Depth Investigation of Archival and Prospectively Collected Samples Reveals No Evidence for XMRV Infection in Prostate Cancer
Source: PLoS One. 2012 Sep 18;7(9):e44954. doi: 10.1371/journal.pone.0044954 (PMC3445615; doi:10.1371/journal.pone.0044954)

**A**

**22RV1 XMRV**

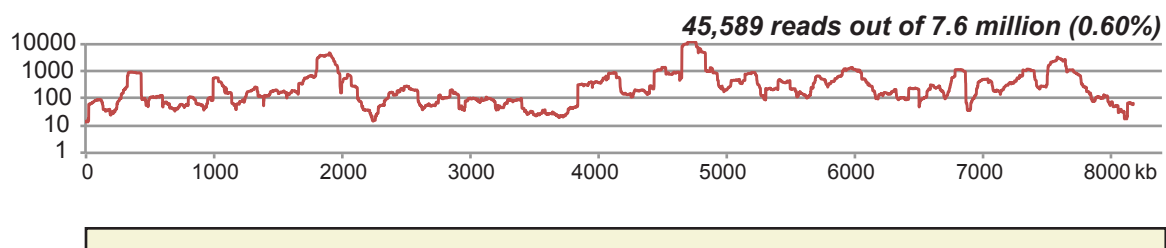

**B**

**MLV-LNCaP**

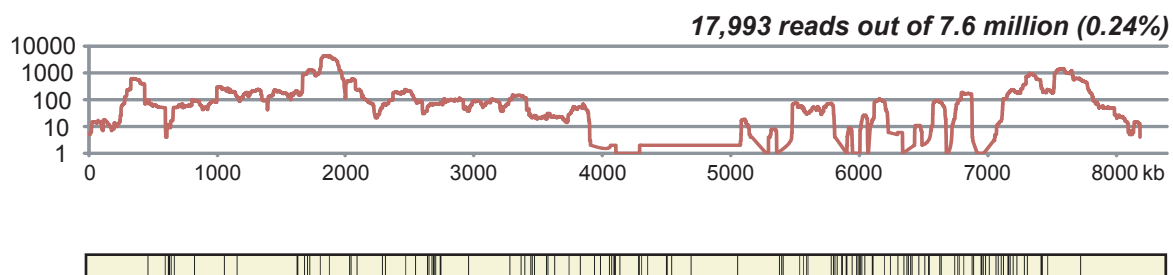

Supplement: Figure S1 — Assembly of Deep Sequencing Reads from XMRV-Infected LNCaP Cells to XMRV and MLV-LNCaP. In 2004, the genome of an MLV related to XMRV (“MLV-LNCaP”) was sequenced from LNCaP cells. In the current study, deep sequencing reads generated from an XMRV-infected 2003 LNCaP cell line were mapped to the genomes of canonical 22Rv1-associated XMRV (GenBank accession number FN692043) and MLV-LNCaP. The 100% identity shared between the consensus XMRV genomes of 2003 LNCaP and 22Rv1 (A), and significant discrepancies between the consensus XMRV genome of 2003 LNCaP and MLV-LNCaP (B) indicate that 22Rv1-associated XMRV, and not MLV-LNCaP, is present in the 2003 LNCaP cells. (PDF) [file pone.0044954.s001.pdf]
