# Supplementary material for: In-Depth Investigation of Archival and Prospectively Collected Samples Reveals No Evidence for XMRV Infection in Prostate Cancer
Source: PLoS One. 2012 Sep 18;7(9):e44954. doi: 10.1371/journal.pone.0044954 (PMC3445615; doi:10.1371/journal.pone.0044954)
Supplement: Table S1 — SNPs in the 22Rv1-Associated XMRV Genome and Comparison to the Prostate Cancer and LNCaP-Associated XMRV Genomes. Approximate p-values are calculated assuming a minimum base quality of 20, or that the reads are >99.0% correct. (PDF) [file pone.0044954.s002.pdf]

Polymorphism	Position	22Rv1 Coverage	22Rv1 Variant Frequency	22Rv1 Variant P-Value	LNCaP Coverage	LNCaP Variant Frequency	VP35 Coverage	VP35 Variant Frequency	VP42 Coverage	VP42 Variant Frequency	VP62 Coverage	VP62 Variant Frequency
G→A	4264	112	38.4%	5.9E-120	128	4.8%	15	20.0%	114	29.8%	25	16.0%
C→G	8112	26	34.6%	7.6E-27	5	0.0%	-	0.0%	33	27.3%	16	25.0%
C→T	3376	90	21.1%	9E-54	142	0.0%	-	0.0%	10	0.0%	3	0.0%
T→C	164	35	11.4%	1.5E-09	10	0.0%	29	0.0%	34	0.0%	-	0.0%
G→C	8100	27	7.4%	0.0000096	6	0.0%	-	0.0%	48	0.0%	19	0.0%
T→C	5580	161	5.0%	9E-16	99	1.0%	20	0.0%	151	0.7%	23	4.3%
T→C	2918	44	4.5%	0.000000067	47	0.0%	17	5.9%	130	1.5%	17	5.9%
T→C	3418	45	4.4%	0.000000033	44	0.0%	3	0.0%	33	0.0%	3	0.0%
T→C	199	25	4.0%	0.00012	13	0.0%	34	0.0%	42	0.0%	1	0.0%
A→G	3546	25	4.0%	0.000097	24	0.0%	-	0.0%	29	0.0%	1	0.0%
G→A	7412	684	3.8%	7.3E-43	369	1.4%	85	0.0%	897	0.2%	206	0.0%
C→T	8042	52	3.8%	0.00000033	22	0.0%	9	0.0%	468	0.4%	110	0.0%
A→G	2282	28	3.6%	0.00025	49	0.0%	11	0.0%	78	0.0%	3	0.0%
T→A	2662	55	3.6%	0.0000008	72	0.0%	19	0.0%	55	0.0%	9	0.0%
G→A	4819	4445	3.6%	6.3E-337	2561	0.9%	74	1.4%	144	0.7%	15	0.0%
A→G	790	58	3.4%	0.00016	60	18.3%	123\	14.6%	459	16.1%	44	13.6%
G→A	1147	65	3.1%	0.0000014	148	0.0%	3	0.0%	24	0.0%	2	0.0%
A→G	3484	32	3.1%	0.00039	21	0.0%	-	0.0%	27	0.0%	1	0.0%
A→G	6517	64	3.1%	0.000083	30	0.0%	29	0.0%	409	0.2%	16	0.0%
