# Supplementary material for: In-Depth Investigation of Archival and Prospectively Collected Samples Reveals No Evidence for XMRV Infection in Prostate Cancer
Source: PLoS One. 2012 Sep 18;7(9):e44954. doi: 10.1371/journal.pone.0044954 (PMC3445615; doi:10.1371/journal.pone.0044954)
Supplement: Table S2 — SNPs in the LNCaP-Associated XMRV Genome and Comparison to the Prostate Cancer and 22Rv1-Associated XMRV Genomes. Approximate p-values are calculated assuming a minimum base quality of 20, or that the reads are >99.0% correct. (PDF) [file pone.0044954.s003.pdf]

Polymorphism	Position	LNCaP Coverage	LNCaP Variant Frequency	LNCaP Variant P-Value	22Rv1 Coverage	22Rv1 Variant Frequency2	VP35 Coverage	VP35 Variant Frequency	VP42 Coverage	VP42 Variant Frequency	VP62 Coverage	VP62 Variant Frequency
C→G	790	60	18.3%	8.60E-31	58	3.4%	123	14.6%	459	16.1%	49	13.6%
G→A	4264	128	14.8%	5.30E-43	112	38.4%	15	20.0%	114	29.8%	25	16.0%
G→T	247	27	11.1%	3.10E-08	80	3.8%	24	4.2%	66	4.5%	1	0.0%
G→A	5551	212	8.5%	3.30E-36	177	3.4%	20	0.0%	231	3.9%	23	0.0%
G→A	2905	100	5.0%	4.40E-15	137	0.7%	17	0.0%	133	3.8%	17	0.0%
T→C	3758	41	4.9%	0.000062	27	0.0%	-	0.0%	19	0.0%	1	0.0%
G→A	3181	90	4.4%	8.20E-10	89	4.5%	-	0.0%	24	4.2%	6	0.0%
T→C	2925	47	4.3%	1.50E-07	43	0.0%	23	4.3%	127	0.0%	18	0.0%
G→A	2830	96	4.2%	8.50E-08	105	0.0%	23	8.7%	158	5.1%	13	0.0%
G→A	2923	48	4.2%	0.000071	43	0.0%	18	0.0%	126	4.8%	17	0.0%
A→G	7943	48	4.2%	2.80E-07	100	1.0%	15	0.0%	161	0.0%	79	0.0%
C→T	3464	25	4.0%	0.00012	29	0.0%	-	0.0%	38	0.0%	1	0.0%
G→A	7916	50	4.0%	0.00012	110	0.0%	30	0.0%	299	0.3%	86	0.0%
A→C	3557	26	3.8%	0.0073	28	0.0%	-	0.0%	29	0.0%	2	0.0%
G→A	6255	160	3.8%	5.50E-12	153	0.7%	5	0.0%	109	2.8%	38	13.2%
G→A	664	54	3.7%	5.80E-08	47	0.0%	65	3.1%	474	2.1%	17	5.9%
G→A	3835	55	3.6%	0.00006	49	0.0%	-	0.0%	23	0.0%	-	0.0%
G→A	5562	138	3.6%	3.90E-09	120	1.7%	21	0.0%	183	1.1%	19	0.0%
C→T	3214	58	3.4%	0.000075	54	0.0%	5	0.0%	57	0.0%	2	0.0%
G→A	3234	58	3.4%	1.3E-09	52	1.9%	3	0.0%	54	9.3%	1	0.0%
G→A	472	60	3.3%	0.00000001	47	4.3%	85	0.0%	125	0.0%	8	0.0%
G→A	3031	90	3.3%	9.4E-09	97	2.1%	7	0.0%	58	1.7%	3	0.0%
G→A	3059	64	3.1%	4.90E-08	86	1.2%	2	0.0%	20	5.0%	4	0.0%
G→A	5703	589	3.1%	1.20E-33	548	2.9%	4	0.0%	118	9.3%	13	0.0%
G→A	5637	100	3.0%	9.1E-10	100	1.0%	28	14.3%	200	0.5%	27	0.0%
